# Sarcopenia Is a Prognostic Factor in Patients Undergoing Percutaneous Endoscopic Gastrostomy

**DOI:** 10.3390/jcm12103360

**Published:** 2023-05-09

**Authors:** Shingo Ono, Hiroto Furuhashi, Shunsuke Kisaki, Hideka Horiuchi, Hiroaki Matsui, Akira Dobashi, Hiroya Ojiri, Kazuki Sumiyama

**Affiliations:** 1 Department of Endoscopy, The Jikei University School of Medicine, 3-25-8 Nishi-Shimbashi, Minato-ku, Tokyo 105-8461, Japan; 2 Department of Radiology, The Jikei University School of Medicine, 3-25-8 Nishi-Shimbashi, Minato-ku, Tokyo 105-8461, Japan

**Keywords:** sarcopenia, L3 skeletal muscle index, percutaneous endoscopic gastrostomy, prognostic factor

## Abstract

(1) Background: Percutaneous endoscopic gastrostomy (PEG) is a widely used long-term enteral nutrition method, but little is known about the associated prognostic factors in patients with PEG. Sarcopenia, a condition characterized by a loss of skeletal muscle mass, increases the risk of developing various gastrointestinal disorders. Yet, the relationship between sarcopenia and the prognosis after PEG remains unclear. (2) Methods: We conducted a retrospective study of patients who underwent PEG consecutively from March 2008 to April 2020. We analyzed preoperative sarcopenia and the prognosis of patients after PEG. We defined sarcopenia as a skeletal muscle index at the level of the third lumbar vertebra of ≤29.6 cm^2^/m^2^ for women and ≤36.2 cm^2^/m^2^ for men. Cross-sectional computed tomography images of skeletal muscle at the level of the third lumbar vertebra were evaluated using DICOM image analysis software (OsiriX). The primary outcome was the difference in overall survival after PEG based on the status of sarcopenia. We also performed a covariate balancing propensity score matching analysis. (3) Results: Of 127 patients (99 men, 28 women), 71 (56%) were diagnosed with sarcopenia, and 64 patients died during the observation period. The median follow-up period did not differ between patients with and without sarcopenia (*p* = 0.5). The median survival time after PEG was 273 days in patients with sarcopenia and 1133 days in those without (*p* < 0.001). Cox proportional hazard model analyses identified three factors that were significantly associated with overall survival: sarcopenia (adjusted hazard ratio [HR]: 2.9, 95% confidence interval [CI]: 1.6–5.4, *p* < 0.001), serum albumin level (adjusted HR: 0.34, 95% CI: 0.21–0.55, *p* < 0.001) and male sex (adjusted HR: 2.0, 95% CI: 1.1–3.7, *p* = 0.03). Propensity score-matched analysis (*n* = 37 vs. 37) showed that the survival rate was lower in the sarcopenia group than in the non-sarcopenia group (at 90 days: 77% (95% CI, 59–88) vs. 92% (76–97), at 180 days: 56% (38–71) vs. 92% (76–97), and at one year: 35% (19–51) vs. 81% (63–91), *p* = 0.0014). (4) Conclusions: Sarcopenia was associated with poor prognosis in patients having undergone PEG.

## 1. Introduction

Percutaneous endoscopic gastrostomy (PEG), which was first described in 1980 [1], has been widely used as a long-term enteral nutrition method for patients with malnutrition due to dysphagia and maintained functional gut. PEG has fewer complications and lower associated mortality than surgical gastrostomy and is a minimally invasive and safe procedure that can be applied even in elderly and debilitated patients [2].

Although the severe complication rate of PEG is low, the procedure is sometimes associated with early mortality. Risk factors associated with post-PEG prognosis may include hypoalbuminemia, a history of aspiration pneumonia, and elevated C-reactive protein levels [3,4,5]. Despite the high number of procedures performed, there is insufficient evidence to identify clear risk factors for poor prognosis in patients undergoing PEG. Knowing these factors at baseline is key to reducing medical procedures and their associated expenses.

Sarcopenia was recognized as an independent condition with an International Classification of Diseases-10 code in 2016 [6]. The prevalence of sarcopenia has increased, and it has become a serious global public health concern for an aging society [7]. Sarcopenia is a syndrome characterized by progressive and generalized loss of skeletal muscle mass and strength with a risk of adverse outcomes such as physical disability, poor quality of life, and poor prognosis. Recently, CT-defined sarcopenia, such as skeletal mass index at the L3 level (L3-SMI), psoas muscle mass index at the L3 level (L3-PMI), and skeletal muscle radiation attenuation at the L3 level (L3-MRA), became known as a prognostic factor in patients with liver diseases and patients after surgery [8,9,10,11,12,13,14]. However, the relationship between sarcopenia and prognosis in PEG patients is still unknown.

In this study, we evaluated whether the existence of sarcopenia at the time of undergoing PEG affects overall survival. We also investigated which of the sarcopenia indices, including L3-SMI, L3-PMI, and L3-MRA, would be the most useful factor for demonstrating the association with poor prognosis.

## 2. Materials and Methods

### 2.1. Study Design

We conducted a retrospective study of patients who consecutively underwent PEG from March 2008 to April 2020 at the Jikei University Hospital (Tokyo, Japan). Patients who had baseline cross-sectional abdominal computed tomography (CT) scans within one month before or two weeks after PEG were included. Exclusion criteria were as follows: (1) advanced pharyngeal, laryngeal, or esophageal cancer; (2) other malignant tumors with palliative therapy; (3) unavailability of cross-sectional CT images at the third lumbar vertebra (L3) level; (4) receiving PEG for a purpose other than nutritional support (i.e., the decompression of the gastrointestinal tract); and (5) lack of clinical data for the analysis (e.g., body height and laboratory data).

### 2.2. Diagnosis of Sarcopenia

**Figure 1:**
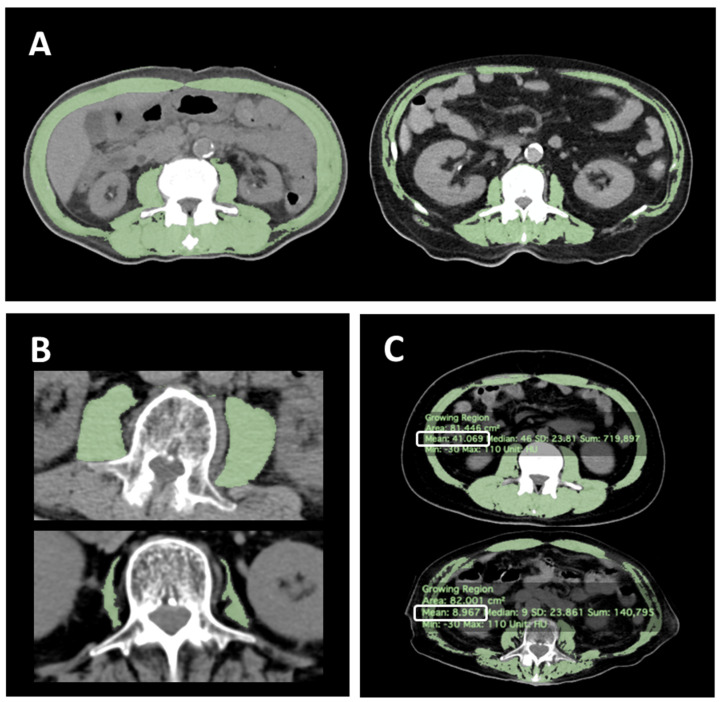
Representative CT images at the level of the third lumbar vertebra. The CT images of patients with (**A**) (**left**) high and (**right**) low L3-SMI and (**B**) (**above**) high and (**below**) low L3-PMI. The areas in green represent the region of skeletal muscle automatically annotated by OsiriX DICOM viewer (version 12.0.3; Pixmeo SARL, Switzerland). (**C**) The images of the skeletal muscles with (**above**) high and (**below**) low values of L3-MRA. The numbers in white squares denote the L3-MRA values that present the average density values (HU) of the skeletal muscle mass at the L3 level. CT, computed tomography; L3-SMI, skeletal muscle mass index at the level of the third lumbar vertebra; L3-PMI, psoas muscle mass index at the level of the third lumbar vertebra; HU, Hounsfield Units; and L3-MRA, muscle radiation attenuation at the level of the third lumbar vertebra.

L3-SMI derived from CT scan was used for the diagnosis of sarcopenia. L3-SMI is a surrogate parameter for evaluating sarcopenia in study participants whose grip strength and walking speed could not be measured (e.g., due to dementia and/or gait disturbance) [15,16]. The patients undergoing PEG at our institution routinely receive preoperative CT scans to verify the anatomical relationship between the stomach and adjacent organs. In this study, a single image that included the spinous process of L3 was collected from the preoperative CT image file for each patient. Then, the skeletal muscle area (SMA) (cm^2^) was automatically quantified within a Hounsfield unit (HU) range of −30 to 110 [17,18,19,20] using OsiriX DICOM viewer (version 12.0.3; Pixmeo SARL, Bernex, Switzerland) after the intra-abdominal organs in that range were manually traced and excluded (Figure 1A). SMA was normalized for body height in meters squared (m^2^) to calculate the lumbar skeletal muscle index (SMI) (cm^2^/m^2^) [21,22,23]. In addition, L3-PMI (psoas muscle area/body height2 [cm^2^/m^2^]) (Figure 1B) and L3-MRA (HU) (Figure 1C) were also evaluated. To diagnose sarcopenia, we used L3-SMI cut-off values of 29.6 cm^2^/m^2^ for women and 36.2 cm^2^/m^2^ for men [24] and L3-PMI cut-off values of 3.92 cm^2^/m^2^ for women and 6.36 cm^2^/m^2^ for men as previously reported [25], whereas the L3-MRA was dichotomized by the median for each gender due to lack of appropriate cut-off values. All measurements were performed by a single trained physician in a blinded manner.

### 2.3. Outcomes

The primary outcome was the difference in overall survival between patients with low and high L3-SMI. Secondary outcomes were (1) the difference in overall survival between patients with low and high L3-PMI, (2) the difference in overall survival between patients with low and high L3-MRA, and (3) the difference in overall survival between patients with low and high L3-SMI in a subgroup with the covariate balancing propensity score matching (CBPS). Primary disease warranting PEG was selected from one of the following: (a) Parkinson’s disease; (b) amyotrophic lateral sclerosis; (c) multiple system atrophy; (d) other neurological disease; (e) cerebral infarction; (f) cerebral hemorrhage; (g) subarachnoid hemorrhage; (h) other cerebrovascular disease; (i) dementia, and (j) other disease.

### 2.4. Statistical Analysis

Between-group differences in demographics and clinical data were evaluated using the Chi square test or Fisher exact test for categorical variables and the Student *t*-test and Mann–Whitney U test for continuous variables. Overall survival was determined using the Kaplan–Meier method. Cox proportional hazards regression model was used to determine the relationship of explanatory variables with overall survival as hazard ratios (HR) and 95% confidence intervals (CI). The cut-off date was set for May 2021. In case of loss to follow-up, the final date the patient was confirmed as alive was censored.

To control for confounding, the following variables were used as the explanatory variables in Cox proportional hazard regression analyses: (1) age (years), (2) sex (male/female), (3) body mass index (BMI) (kg/m^2^]), (4) serum albumin level (g/dL), (5) C-reactive protein (mg/dL), (6) total lymphocyte count [26], and (7) previous history of pneumonia (yes/no). Continuous variables were categorized into binary variables: <1.0 and ≥1.0 (mg/dL) for C-reactive protein (4) and <1.5 and ≥1.5 (×10^9^/L) for total lymphocyte count [26]. Bayesian information criterion was used for model selection. CBPS was performed using ‘CBPS’ package in R using nearest-neighbor matching (1:1 ratio) with a caliper width of 0.2 of the SD of the logit of the score. Matched variables were (1) age (years), (2) sex (male/female), (3) serum albumin level (g/dL), (4) C-reactive protein (mg/dL), (5) total lymphocyte count (TLC) (mg/dL), and (6) previous history of pneumonia (yes/no).

Log-rank test was applied to compare overall survival between groups after matching. Sensitivity analysis was performed for the cut-off values, binarization of variables, the method selecting explanatory variables used for multivariate analysis, and survival analysis when propensity score was used as a covariate instead of matching. Two-tailed tests were used to compare two groups, and *p* < 0.05 was considered significant. All statistical analyses and graphing were carried out using R version 4.0.2 (R Foundation for Statistical Computing, Vienna, Austria) online.3.

## 3. Results

### 3.1. Patients Enrollments

**Figure 2:**
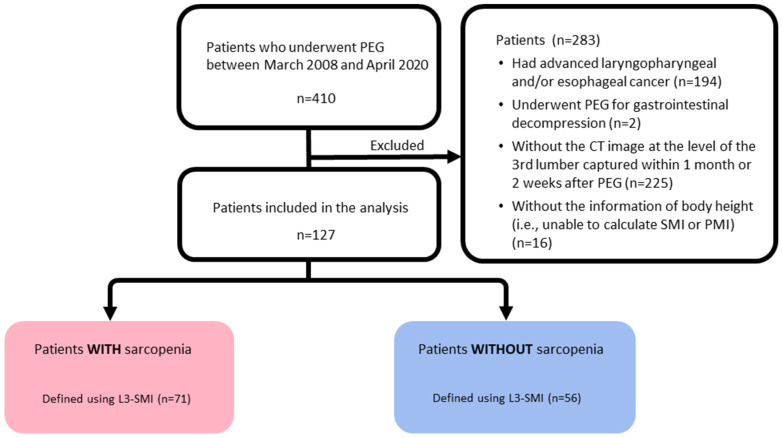
Flow diagram of the study selection process.

Of the 410 patients who underwent PEG during the study period, 283 met at least one of the exclusion criteria, so 127 were selected for analysis. Of these 127 patients, 71 cases (56%) were classified as low L3-SMI, 101 cases (80%) as low L3-PMI, and 63 cases (50%) as low L3-MRA (Figure 2).

PEG, percutaneous endoscopic gastroplasty; CT, computed tomography; L3-SMI, skeletal muscle mass index at the level of the third lumbar vertebra; L3-PMI, psoas muscle mass index at the level of the third lumbar vertebra; and L3-MRA, skeletal muscle radiation attenuation at the level of the third lumbar vertebra.

### 3.2. Patients Characteristics

Table 1 shows the characteristics of all patients and each group based on the status of L3-SMI, L3-PMI, and L3-MRA. The low L3-SMI group was significantly older (*p* = 0.04) and had lower BMI (*p* < 0.001) compared with the high L3-SMI group. There was no difference in albumin levels between these two groups (*p* = 0.2). Men (*p* = 0.001) were significantly more represented in the low L3-PMI group. The high L3-PMI group had significantly lower albumin levels (*p* = 0.03), cholinesterase (*p* = 0.002), and platelets (*p* = 0.002).

### 3.3. Overall Follow-Up Period and Events

The median follow-up period was 716 (502–941) days, during which 64 patients (50.4%) died. The median overall survival was 666 (387–992) days. The overall survival rate was 95.2% (89.7–97.8) at 30 days, 90.3% (83.6–94.4) at 60 days, 83.6% (75.7–89.1) at 90 days, and 60.1% (50.3–68.6) at 180 days. The median follow-up period did not differ significantly between the low and high groups in each of the three indices: L3-SMI, 645 (356–945) vs. 811 (411–972) days (*p* = 0.5); L3-PMI, 889 (455–1240) vs. 694 (411–941) days (*p* = 0.6); and 582 (52–NA) vs. 943 (688–1378) days (*p* = 0.052).

Of the 64 deaths, 46 deaths were observed in the low L3-SMI group, and 18 deaths were observed in the high L3-SMI group. Among the 46 deaths in the low L3-SMI group, the cause of death was identified in 38 patients. The most frequent cause of death was pneumonia (*n* = 29), and the other deaths were due to cardiovascular disease (*n* = 2), renal failure (*n* = 1), gastrointestinal necrosis (*n* = 1), superior mesenteric artery embolism (*n* = 1), and death due to primary disease (*n* = 4). Among the 18 deaths in the high L3-SMI group, the cause of death was identified in 13 patients. Six patients died of pneumonia. Other deaths were due to cardiovascular disease (*n* = 2), hepatic failure (*n* = 1), gastrointestinal bleeding (*n* = 1), and death of primary disease (*n* = 3). No PEG-related death was observed in either group. The pneumonia mortality rate tended to be higher in the Low L3-SMI group though the difference was not statistically significant (Low SMI group vs. High SMI group = 76.3% vs. 46.2%; *p* = 0.08).

### 3.4. Survival Rates and Log-Rank Analyses at 90 and 180 Days and One Year

**Figure 3:**
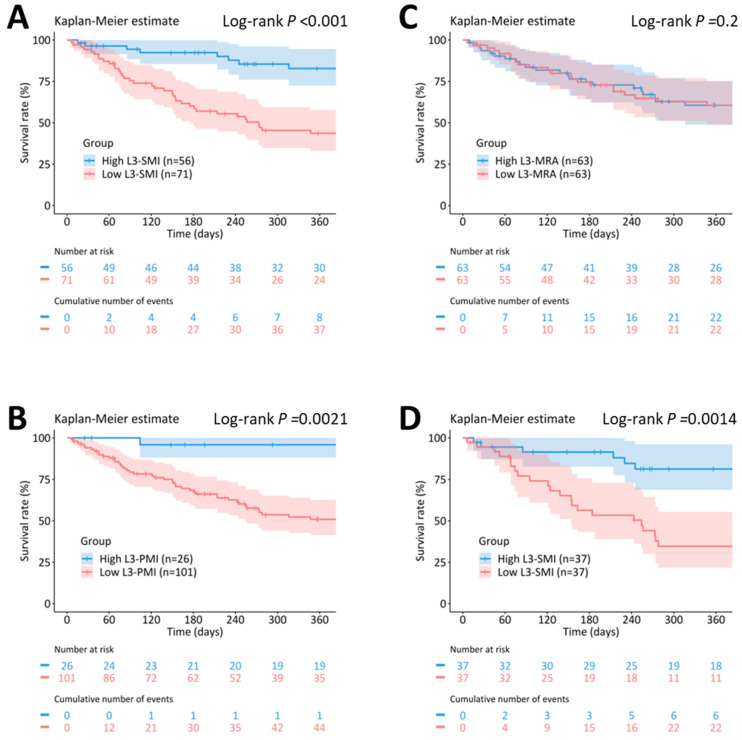
Kaplan–Meier survival curves in patients with and without sarcopenia. Kaplan–Meier survival curves for (**A**) High L3-SMI and Low L3-SMI, (**B**) High L3-PMI and Low L3-PMI, (**C**) High L3-MRA and Low L3-MRA, and (**D**) High L3-SMI and Low L3-SMI after covariate balancing propensity score matching.

The median survival was significantly shorter in the low SMI group than in the high SMI group: 273 (95% CI, 163–638) vs. 1133 (666–NA) days; *p* < 0.001 (Figure 3A). The survival rate at 90 days was 75.4% (63.4–83.9) in the low L3-SMI group vs. 94.4% (83.6–98.2) in the high L3-SMI group, 60.1% (47.4–70.7) vs. 92.4% (80.9–97.1) at 180 days and 43.7% (31.3–55.3) vs. 82.8% (68.2–91.1) at one year of follow-up.

The median survival was significantly shorter in the low PMI group than in the high PMI group: 387 (245–863) vs. 1133 (736–NA) days; *p* = 0.0021 (log-rank test) (Figure 3B). The survival rate at 90 days was 79.4% (69.9–86.2) in the low L3-PMI vs. 100% (NA–NA) in the high L3-PMI group, 68.4% (58.0–76.8) vs. 95.8% (73.9–99.4) at 180 days, and 50.9% (39.9–60.9) vs. 95.8% (73.9–99.4) at one year.

The overall survival did not differ significantly between the low and high MRA group (median survival days, 638 (278–867) vs. 1133 (274–NA) days; *p* = 0.2 (log-rank test)) (Figure 3C). We observed no difference between the two groups in the 90-day survival rate (85.0% (73.2–91.9) vs. 83.5% (71.5–90.8)), 180-day survival rate (74.5% (61.3–83.8) vs. 74.7% (61.5–83.9)) or the one-year survival rate (60.6% (46.3–72.2) vs. 60.6% (46.3–72.2)).

Sarcopenia was defined by (A) L3-SMI, (B) L3-PMI, and (C) L3-MRA in all patients (*n* = 127). (D) Subgroup analysis with propensity score matching of known prognostic predictors (high vs. low L3-SMI) (*n* = 74). The color bands represent a 95% confidence interval in each group. L3-SMI, skeletal muscle mass index at the level of the third lumbar vertebra; L3-PMI, psoas muscle mass index at the level of the third lumbar vertebra; and L3-MRA, skeletal muscle radiation attenuation at the level of the third lumbar vertebra.

### 3.5. Univariate and Multivariate Cox Proportional Hazard Analyses

In univariate analyses, L3-SMI, L3-PMI, and all known risk factors (male sex, age, BMI, hypoalbuminemia, total lymphocyte count < 1.5 × 10^9^/L, C-reactive protein ≥ 1.0 mg/dL and underlying pneumonia) emerged as predictive factors related to poor prognosis (Table 2). In multivariate analyses, low L3-SMI, male sex, and hypoalbuminemia were independent risk factors for poor overall survival.

### 3.6. Covariate-Balancing Propensity Score Matching Analyses

Based on the covariate balancing propensity scores, the two groups were matched using the covariates of age, gender, BMI, the coexistence of pneumonia, albumin level, CRP, and lymphocyte count, and 37 patients were selected from each group. Most of the standardized mean differences for the matched factors decreased after matching (Table 3). The log-rank analysis revealed that the overall survival was significantly shorter in the low SMI group (254 (95% CI, 124–538) vs. 1341 (604–NA) days; *p* = 0.0014) (Figure 3D). The survival rate at 90 days was 77.1% (59.3–87.8) in the low L3-SMI group vs. 91.5% (75.8–97.2) in the L3-SMI group, 56.3% (38.3–71.0) vs. 91.5% (75.8–97.2) at 180 days, and 34.7% (19.2–50.6) vs. 81.3% (62.9–91.2) at one year of follow-up.

### 3.7. Correlation between Sarcopenia-Related Indices and Relationship with Body Mass Index

**Figure 4:**
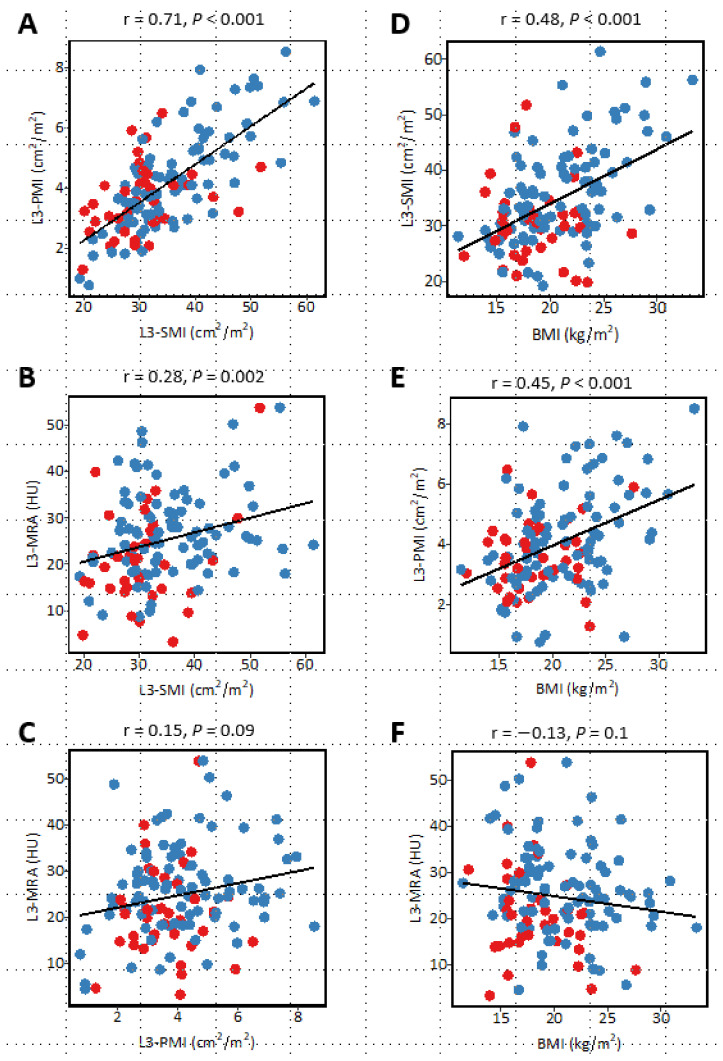
Correlations between each sarcopenia-related indices and between each index and body mass index. Scatterplots for (**A**) L3-SMI and L3-PMI, (**B**) L3-SMI and L3-MRA, and (**C**) L3-PMI and L3-MRA, (**D**) BMI and L3-SMI, (**E**) BMI and L3-PMI, and (**F**) BMI and L3-MRA. Red dots denote female patients, and blue dots show male patients. The black line represents a linear regression line; the region in gray denotes 95% CIs. L3-SMI, skeletal muscle mass index at the level of the third lumbar vertebra. L3-PMI, psoas muscle mass index at the level of the third lumbar vertebra; and L3-MRA, skeletal muscle radiation attenuation at the level of the third lumbar vertebra; and BMI, body mass index.

The correlation coefficient between sarcopenia-related indices and BMI is described in Figure 4. A very strong correlation was observed between L3-SMI and L3-PMI (r = 0.71, *p* < 0.001), and each of them showed a moderate correlation with BMI (L3-SMI, r = 0.48, *p* < 0.001; L3-PMI, r = 0.45, *p* < 0.001; L3-MRA, r = −0.13, *p* = 0.1). This trend was observed irrespective of gender. Meanwhile, the relationships between L3-MRA and L3-SMI, L3-PMI, and BMI, respectively, was weak (L3-SMI, r = 0.28, *p* = 0.002; L3-PMI, r = 0.15, *p* = 0.09; BMI, r = −0.13, *p* = 0.1) (Figure 4).

## 4. Discussion

To the best of our knowledge, this is the first study to identify preoperative sarcopenia as a prognostic factor for poor survival after PEG in geriatric patients. Hypoalbuminemia was previously established as a prognostic factor associated with PEG, but in this study, sarcopenia emerged as a new prognostic factor associated with PEG independent of hypoalbuminemia.

In the literature, sarcopenia and prognosis have been positively and negatively associated. CT-defined sarcopenia has been known as a prognostic predictor of several hepatic diseases [13,24,27,28,29,30], yet sarcopenia does not always worsen survival [31,32,33]. In our study, sarcopenia negatively affected the prognosis in patients who underwent PEG.

Although there was no significant difference observed in the univariate analysis of L3-SMI with respect to the presence of underlying pneumonia (Supplementary Table S1), pneumonia-related death tended to be more frequent in patients with sarcopenia. Sarcopenia has been identified as a risk factor for pneumonia because of poor chewing and swallowing functions, delayed mobilization, dysphagia, or difficulty in clearing the airway [34]. Sarcopenia is also associated with reduced glutamine production, leading to intestinal dysfunction and infectious complications [35]. These factors may be related to higher mortality related to pneumonia after PEG in patients with sarcopenia.

In this study, among the indicators of sarcopenia, L3-SMI showed significant differences in multivariate analysis. We only diagnosed sarcopenia by skeletal muscle mass in CT images and did not evaluate other factors of sarcopenia. Grip strength and walking speed are commonly used as diagnostic indicators for sarcopenia, but it would be difficult to measure such muscle function in PEG patients because of their severe frailty. However, the correlation between L3-SMI and grip strength has been already reported [36], and L3-SMI has been discerned as an important indicator of sarcopenia. Thus, it was appropriate to use CT images, which are objective and usually taken preoperatively for patients with PEG, for the evaluation of sarcopenia in our study. L3-SMI may be the most suitable factor for predicting prognosis, especially for evaluating sarcopenia in PEG patients.

Median survival did not differ significantly between the low and high L3-MRA groups in this study. A recent study concluded that low L3-MRA, which equally demonstrates sarcopenic obesity, was also associated with higher mortality in patients with hepatocellular carcinoma [24], but we could not confirm that observation. The reason L3-MRA did not affect prognosis in our study may be the limitations of assessing L3-MRA in PEG patients. Since L3-MRA is a method for evaluating tissue components using the CT value of skeletal muscle, it may fluctuate due to the influence of the CT value. In cases with strong edematous changes, as in PEG patients who may have constitutional edema associated with hypoalbuminemia, the CT value in the muscle is expected to decrease due to an increase in water content, and this might affect the L3-MRA level in our study.

In order to minimize the influence of potential confounding variables, we evaluated the prognostic impact of preoperative sarcopenia using CBPS and sensitivity analysis. This analysis provided additional evidence that can support and strengthen the relationship between sarcopenia and poor prognosis and demonstrated that sarcopenia was a robust prognostic factor after PEG compared to other known prognostic factors (Supplementary Table S2).

From our result, assessing sarcopenia in addition to existing independent prognostic factors of PEG may provide important information for patients and their families to discuss the indication of PEG. Evaluating sarcopenia at baseline may lead to the development of tailored preventive strategies, such as early nutritional interventions, physical exercise programs, and individualized care plans for patients with sarcopenia who undergo PEG placement.

This study has limitations. 1. The major population of PEG in the present study was non-malignancy patients with dysphagia (such as Parkinson’s disease, amyotrophic lateral sclerosis, multiple system atrophy, and other neurological diseases). We excluded malignancy patients in this study because prognosis can vary depending on cancer stage classification in malignancy patients. Our result may only be limited to non-malignancy patients. 2. Primary and secondary sarcopenia were not distinguished in this study. The results may differ if they were considered separately. Although most of the patients were considered to have secondary sarcopenia associated with malnutrition, low activity, and diseases, it was difficult to distinguish them retrospectively. 3. We excluded patients without pre-procedural CT in this study. It was unclear why procedural CT was not performed in these patients. Although the patients’ BMI and survival were similar between patients with and without pre-procedural CT (BMI: 19.7 vs. 19.3, *p* = 0.3, survival days: 384 days vs. 416 days, *p* = 0.5), this exclusion may introduce bias in patient selection. 4. We only included cases with plain CT, as skeletal muscle measurements could be influenced by the use of contrast agent in contrast CT, which may result in selection bias [37]. 5. The cut-off value may not be appropriate in this study. We used the previously reported Asian cut-off value because an association of sarcopenia with PEG patients has not been reported. Since the general condition and nutritional status of patients undergoing PEG tend to be poor, new cut-off values for defining sarcopenia in PEG patients may be considered.

## 5. Conclusions

In conclusion, the presence of preoperative sarcopenia was identified as an independent prognostic factor for patients undergoing PEG. Assessing sarcopenia may provide important information for the indication of PEG and may lead to the development of tailored preventive strategies in an increasingly aging society.

## Figures and Tables

**Table 1:** Difference in background characteristics of the patients in high and low groups of L3-SMI, L3-PMI, and L3-MRA.

		L3-SMI					L3-PMI					L3-MRA				
Factor		High		Low		*p* Value	High		Low		*p* Value	High		Low		*p* Value
*n*		56		71			26		101			63		64		
Sex, *n* (%)	Female	20	(35.7)	18	(25.4)	0.2	15	(57.7)	23	(22.8)	0.001	19	(30.1)	18	(28.1)	1
	Male	36	(64.3)	53	(74.6)		11	(42.3)	78	(77.2)		44	(70.0)	46	(71.9)	
Age, mean (SD)	years	70.0	(14.3)	75.4	(14.5)	0.04	71.0	(11.5)	73.5	(15.3)	0.4	71.8	(15.3)	74.2	(13.9)	0.4
Body height, mean (SD)	cm	159.9	(9.3)	161.1	(9.1)	0.5	157.1	(7.6)	161.5	(9.4)	0.03	160.4	(8.4)	161.1	(10.0)	0.7
Body weight, mean (SD)	kg	56.1	(15.2)	49.9	(11.6)	0.01	52.8	(14.1)	52.6	(13.6)	0.9	50.0	(12.2)	55.3	(14.6)	0.03
BMI, mean (SD)	kg/m^2^	21.7	(4.3)	19.1	(3.7)	<0.001	21.2	(4.9)	20.0	(3.4)	0.2	19.2	(3.7)	21.2	(4.4)	0.01
Pneumonia, *n* (%)	No	38	(67.9)	34	(47.9)	0.03	18	(69.2)	54	(53.5)	0.2	33	(52.4)	38	(60.3)	0.5
	Yes	18	(32.1)	37	(52.1)		8	(30.8)	47	(46.5)		30	(47.6)	25	(39.7)	
Total protein, mean (SD)	g/dL	6.4	(0.8)	6.3	(0.8)	0.6	6.5	(0.6)	6.3	(0.8)	0.1	6.4	(0.8)	6.2	(0.8)	0.4
Serum albumin, mean (SD)	g/dL	3.0	(0.6)	2.9	(0.6)	0.2	3.2	(0.5)	2.9	(0.6)	0.03	3.0	(0.6)	2.8	(0.6)	0.04
Serum TC, mean (SD)	mg/dL	48.8	(90.6)	64.5	(84.6)	0.5	47.8	(71.8)	60.0	(89.1)	0.7	43.6	(71.3)	80.6	(102.2)	0.09
Serum ChE, mean (SD)	U/L	201	(743.7)	183.1	(80)	0.2	2364	(69)	180.8	(76)	0.002	198	(78)	183.2	(77)	0.3
Serum CRP, mean (SD)	mg/dL	1.2	(2.2)	1.8	(2.4)	0.2	1.1	(1.6)	1.6	(2.4)	0.3	1.1	(1.7)	1.9	(2.7)	0.04
PNI, mean (SD)		36.5	(6.9)	35.7	(7.6)	0.5	38.3	(5.6)	35.5	(7.6)	0.09	36.9	(7.1)	35.1	(7.4)	0.2
Hemoglobin, mean (SD)	g/dL	11.7	(1.8)	11.1	(2.0)	0.08	11.9	(1.7)	11.2	(1.9)	0.1	11.7	(1.9)	11.0	(1.9)	0.03
Platelet, mean (SD)	×10^9^/L	261	(121)	233	(106)	0.2	306	(146)	230.1	(99)	0.002	258	(134)	230.9	(87)	0.2
WBC, mean (SD)	×10^9^/L	6.4	(2.0)	9.4	(21.8)	0.3	6.8	(1.7)	8.4	(18.3)	0.7	6.4	(2.4)	9.7	(23.0)	0.3
TLC, mean (SD)	×10^9^/L	1.3	(0.5)	1.4	(0.7)	0.3	1.4	(0.5)	1.4	(0.7)	0.8	1.3	(0.6)	1.4	(0.7)	0.5

L3-SMI, skeletal muscle mass index at the third lumbar level; L3-PMI, psoas muscle mass index at the third lumbar level; L3-MRA, skeletal muscle radiation attenuation at the third lumbar level; SD, standard deviation; BMI, body mass index; TC, total cholesterol; ChE, Choline esterase; CRP, C-reactive protein; PNI, Onodera’s prognostic nutritional index; WBC, white blood cell count; and TLC, total lymphocyte count.

**Table 2:** Results of univariate and multivariate Cox proportional hazard regression analysis.

	Univariate Cox Proportional Hazard Regression Analysis		Multivariate Cox Proportional Hazard Regression Analysis *	
Variables	HR (95% CI)	*p* Value	Adjusted HR (95% CI)	*p* Value
L3-SMI, low group	2.8 (1.6–4.8)	<0.001	2.9 (1.6–5.4)	<0.001
L3-PMI, low group	3.2 (1.5–7.1)	0.004	(-)	
L3-MRA, low group	1.4 (0.9–2.4)	0.2	(-)	
Sex, male	1.8 (1.0–3.3)	0.046	2.0 (1.1–3.7)	0.03
Age, per year	1.9 (1.2–3.2)	0.01	(-)	
BMI, per kg/mm^2^	0.94 (0.89–1.0)	0.049	(-)	
Serum albumin, per g/dL	0.34 (0.21–0.56)	<0.001	0.34 (0.21–0.55)	<0.001
TLC, <1.5 ×10^9^/L	1.8 (1.0–3.0)	0.04	(-)	
Serum CRP, ≥1.0 mg/dL	2.5 (1.5–4.1)	0.0004	(-)	
Underlying pneumonia, yes	1.9 (1.1–3.2)	0.02	(-)	

* In the multivariate analysis, explanatory variables were selected using Bayesian information criterion. HR, hazard ratio; CI, confidence interval; L3-SMI, skeletal muscle mass index at the third lumber level; L3-PMI, Psoas muscle mass index at the third lumber level; L3-MRA, Muscle radiation attenuation at the third lumber level; BMI, body mass index; CRP, C-reactive protein; PNI, Onodera’s prognostic nutritional index; WBC, white blood cell count; and TLC, total lymphocyte count.

**Table 3:** Difference in background characteristics of the patients between low and high L3-SMI group before- and after-covariate balancing propensity score matching.

		Before-Matching					After-Matching				
Factor		High L3-SMI		Low L3-SMI		SMD	High L3-SMI		Low L3-SMI		SMD
*n*		56		71			37		37		
Matched variables											
Sex, *n* (%)	Female	20	(35.7)	18	(25.4)	0.226	13.0	(35.1)	11.0	(29.7)	0.116
	Male	36	(64.3)	53	(74.6)		24.0	(64.9)	26.0	(70.3)	
Age, mean (SD)	years	70.0	(14.3)	75.4	(14.5)	0.374	73.1	(13.0)	73.2	(17.5)	0.009
BMI, mean (SD)		21.7	(4.3)	19.1	(3.7)	0.644	21.3	(4.7)	20.6	(4.1)	0.15
Pneumonia, *n* (%)	No	38	(67.9)	34	(47.9)	0.413	22.0	(59.5)	24.0	(64.9)	0.112
	Yes	18	(32.1)	37	(52.1)		15.0	(40.5)	13.0	(35.1)	
Albumin, mean (SD)	g/dL	3.0	(0.6)	2.9	(0.6)	0.235	2.9	(0.6)	3.0	(0.7)	0.072
CRP, mean (SD)	mg/dL	1.2	(2.2)	1.8	(2.4)	0.257	1.3	(2.5)	1.4	(2.4)	0.075
TLC, mean (SD)	×10^9^/L	1.3	(0.5)	1.4	(0.7)	0.176	1364.9	(529.8)	1224.3	(530.4)	0.265
Non-matched variables											
Body height, mean (SD)	cm	159.9	(9.3)	161.1	(9.1)	0.134	8.8	(8.8)	161.6	(8.7)	0.341
Body weight, mean (SD)	kg	56.1	(15.2)	49.9	(11.6)	0.462	15.2	(15.2)	54.0	(12.7)	0.005
Total protein, mean (SD)	g/dL	6.4	(0.8)	6.3	(0.8)	0.098	1.0	(0.9)	6.2	(0.7)	0.146
PNI, mean (SD)		36.5	(6.9)	35.7	(7.6)	0.114	6.9	(6.9)	35.6	(7.8)	0.033
ChE, mean (SD)	U/L	201.8	(73.7)	183.1	(79.6)	0.244	64.1	(64.1)	200.1	(88.5)	0.173
WBC, mean (SD)	×10^9^/L	6.4	(2.0)	9.4	(21.8)	0.193	1842.9	(1842.9)	11,329.7	(30,116.8)	0.241
Hemoglobin, mean (SD)	g/dL	11.7	(1.8)	11.1	(2.0)	0.315	1.8	(1.7)	11.0	(2)	0.182
Platelet count, mean (SD)	×10^9^/L	261.7	(121.4)	233.0	(105.8)	0.252	13.7	(13.7)	23.8	(8.1)	0.299

L3-SMI, skeletal muscle mass index at the third lumber level; SMD, standardized mean difference; SD, standard deviation; BMI, body mass index; CRP, C-reactive protein; PNI, Onodera’s prognostic nutritional index; WBC, white blood cell count; and TLC, total lymphocyte count.

## Data Availability

The data that support the findings of this study are available from the corresponding author upon reasonable request.

## Supplementary Materials

The following supporting information can be downloaded at: https://www.mdpi.com/article/10.3390/jcm12103360/s1, Table S1: Difference in background characteristics of the patients with or without underlying pneumonia, Table S2: Sensitivity analysis.
