# Supplementary material for: Sarcopenia Is a Prognostic Factor in Patients Undergoing Percutaneous Endoscopic Gastrostomy
Source: J Clin Med. 2023 May 9;12(10):3360. doi: 10.3390/jcm12103360 (PMC10218806; doi:10.3390/jcm12103360)
Supplement: Supplementary file 1 [file jcm-12-03360-s001.zip › jcm-2250572-supplementary.pdf]

Supplementary Table S1

Difference in background characteristics of the patients with or without underlying pneumonia

Factor		Underlying pneumonia: Yes	Underlying pneumonia: No	*P* value
N		55	72	
Sex, n (%)	Female	13 (23,6)	25 (34.7)	0.06
	Male	42 (76.4)	47 (65.3)	
Age, mean (SD)	years	77.4 (12.3)	69.6 (15.5)	0.003
BMI, mean (SD)	kg/m^2	19.1 (3.9)	20.9 (4.1)	0.02
L3-SMI, mean (SD)		32.9 (7.9)	34.2 (10.2)	0.4
Total protein, mean (SD)	g/dL	6.3 (0.7)	6.3 (0.9)	0.6
Serum albumin, mean (SD)	g/dL	2.74 (0.6)	3.06 (0.6)	0.002
Serum ChE, mean (SD)	U/L	165 (55)	213 (83)	<0.001
Serum CRP, mean (SD)	mg/dL	1.7 (2.0)	1.4 (2.5)	0.5
PNI, mean (SD)		34.3 (6.8)	37.5 (7.4)	0.01
Hemoglobin, mean (SD)	g/dL	11.1 (1.8)	11.5 (2.0)	0.2
Platelet, mean (SD)	$\times 10^9$/L	22.9 (7.2)	25.8 (13.7)	0.2
WBC, mean (SD)	$\times 10^9$/L	6.6 (2.5)	6.8 (2.6)	0.7
TLC, mean (SD)	$\times 10^9$/L	1.4 (0.7)	1.4 (0.6)	0.9

L3-SMI, skeletal muscle mass index at the third lumbar level; SD, standard deviation; BMI, body mass index; TC, total cholesterol; ChE, Choline esterase; CRP, C-reactive protein; PNI, Onodera's prognostic nutritional index; WBC, white blood cell count; and TLC, total lymphocyte count.

Supplementary Table S2

A. Sensitivity analysis for the cut-off values of L3-SMI

Iritani et al. [J Gastroenterol 2015;50(3):323-332] proposed different L3-SMI cut-off values in Japanese individuals (≤ 29.0 cm^2/m^2 for women; ≤ 36.0 cm^2/m^2 for men). The Cox proportional hazard model analysis based on Iritani's cut-off value showed that low L3-SMI remained an independent risk factor for short survival as well as hypoalbuminemia.

	Univariate analysis		Multivariate analysis (BIC) *		Multivariate analysis (AIC) †	
Variables	HR (95% CI)	*P* value	Adjusted HR (95% CI)	*P* value	Adjusted HR (95% CI)	*P* value
L3-SMI, low (Iritani's cut-off value)	2.6 (1.6 – 4.4)	<0.001	2.6 (1.5 – 4.4)	<0.001	2.5 (1.4 – 4.2)	0.0013
Sex, male	1.8 (1.0 – 3.3)	0.046	(-)		1.9 (1.0 – 3.5)	0.047
Age, per year	1.9 (1.2 – 3.2)	0.01	(-)		(-)	
BMI, per kg/m^2	0.94 (0.89 – 1.0)	0.049	(-)		(-)	
Serum albumin, per g/dL	0.34 (0.21 – 0.56)	<0.001	0.35 (0.21 – 0.57)	<0.001	0.36 (0.22 – 0.60)	<0.001
TLC, <1.5 $\times 10^9$/L	1.8 (1.0 – 3.0)	0.040	(-)		1.6 (0.91 – 2.7)	0.10
Serum CRP, ≥1.0 mg/dL	0.34 (0.21 – 0.56)	<0.001	(-)		(-)	
Underlying pneumonia	1.9 (1.1 – 3.2)	0.02	(-)		(-)	

* Explanatory variables were selected using Bayesian information criterion. HR, hazard ratio; † Explanatory variables were selected using Akaike information criterion. BIC, Bayesian information criterion; AIC, Akaike information criterion; HR, hazard ratio; CI, confidence interval; L3-SMI, Skeletal muscle mass index at the third lumber level; BMI, body mass index; CRP, C-reactive protein; PNI, Onodera's prognostic nutritional index; WBC, white blood cell count; and TLC, total lymphocyte count.

B. Sensitivity analysis for the binarization of the explanatory variables

In the main analyses, we converted the continuous variables of 'total lymphocyte count' and 'C-reactive protein' to binary variables according to a previous study, whereas albumin level was incorporated into the model as a continuous variable. The optimal cut-off values for binarization in our cohort, however, were not known, and several have suggested binarizing albumin with a cut-off value of 2.5 g/dL. Here, we performed a Cox proportional hazard analysis (a) without and, conversely, (b) with binarizing albumin level. The results suggested that low L3-SMI remained an independent risk factor for short survival regardless of the binarization of the explanatory variables.

(a) Cox proportional hazard analysis without binarizing continuous variables

	Univariate analysis		Multivariate analysis (BIC)		Multivariate analysis (AIC)	
Variables	HR (95% CI)	*P* value	Adjusted HR (95% CI)	*P* value	Adjusted HR (95% CI)	*P* value
L3-SMI, low	2.8 (1.6 – 4.8)	<0.001	3.0 (1.7 – 5.2)	<0.001	3.1 (1.7 – 5.4)	<0.001
Sex, male	1.8 (1.0 – 3.3)	0.046	2.0 (1.1 – 3.7)	0.03	2.0 (1.1 – 3.7)	0.031
Age, per year	1.9 (1.2 – 3.2)	0.019	(-)		(-)	
BMI, per kg/m2	0.94 (0.89 – 1.0)	0.049	(-)		(-)	
Serum albumin, per g/dL	0.34 (0.21 – 0.56)	<0.001	0.34 (0.21 – 0.55)	<0.001	0.36 (0.22 – 0.60)	<0.001
TLC, per /µL	1.0 (1.0 – 1.0)	0.11	(-)		1.0 (1.0 – 1.0)	0.15
Serum CRP, per mg/dL	1.1 (1.1 – 1.2)	0.0010	(-)		(-)	
Underlying pneumonia	1.9 (1.1 – 3.2)	0.018	(-)		(-)	

* Explanatory variables were selected using Bayesian information criterion. HR, hazard ratio; † Explanatory variables were selected using Akaike information criterion. BIC, Bayesian information criterion; AIC, Akaike information criterion; HR, hazard ratio; CI, confidence interval; L3-SMI, Skeletal muscle mass index at the third lumber level; BMI, body mass index; CRP, C-reactive protein; PNI, Onodera's prognostic nutritional index; WBC, white blood cell count; and TLC, total lymphocyte count.

(b) Cox proportional hazard analysis with binarizing the serum albumin level

	Univariate analysis		Multivariate analysis (BIC) *		Multivariate analysis (AIC) †	
Variables	HR (95% CI)	*P* value	Adjusted HR (95% CI)	*P* value	Adjusted HR (95% CI)	*P* value
L3-SMI, low	2.8 (1.6 – 4.8)	<0.001	3.2 (1.8 – 5.7)	<0.001	2.9 (1.6 – 5.1)	<0.001
Sex, male	1.8 (1.0 – 3.3)	0.046	(-)		1.8 (0.92 – 3.3)	0.091
Age, per year	1.9 (1.2 – 3.2)	0.01	(-)		1.0 (1.0 – 1.0)	0.11
BMI, per kg/m^2	0.94 (0.89 – 1.0)	0.049	(-)		(-)	
Serum albumin, <2.5 mg/dL	2.6 (1.5 – 4.5)	<0.001	(-)		1.9 (0.98 – 3.5)	0.057
TLC, <1.5 $\times 10^9$/L	1.8 (1.0 – 3.0)	0.040	2.1 (1.1 – 3.8)	0.019	1.6 (0.90 – 2.8)	0.11
Serum CRP, ≥1.0 mg/dL	0.34 (0.21 – 0.56)	<0.001	2.2 (1.3 – 3.9)	0.0059	1.9 (1.0 – 3.5)	0.047
Underlying pneumonia	1.9 (1.1 – 3.2)	0.02	(-)		(-)	

* Explanatory variables were selected using Bayesian information criterion. HR, hazard ratio; † Explanatory variables were selected using Akaike information criterion. HR, hazard ratio; CI, confidence interval; L3-SMI, Skeletal muscle mass index at the third lumber level; BMI, body mass index; CRP, C-reactive protein; PNI, Onodera's prognostic nutritional index; WBC, white blood cell count; and TLC, total lymphocyte count.

C. Sensitivity analysis for the selection method for explanatory variables

As primary analyses, we applied the Bayesian information criterion as the selection method for explanatory variables. Here, we performed variable selection methods using **(a)** Akaike information criterion, and *P*-value in univariate analysis of under **(b)** 0.1, **(c)** 0.05, and **(d)** 0.01. The results suggested that low L3-SMI remained an independent risk factor for short survival regardless of the selection method.

(a) Cox proportional hazard analysis with variable selection method using Akaike information criterion

	Univariate analysis		Multivariate analysis (AIC)	
Variables	HR (95% CI)	*P* value	Adjusted HR (95% CI)	*P* value
L3-SMI, low	2.8 (1.6 – 4.8)	<0.001	2.9 (1.6 – 5.1)	<0.001
Sex, male	1.8 (1.0 – 3.3)	0.046	2.0 (1.1 – 3.7)	0.030
Age, per year	1.9 (1.2 – 3.2)	0.01	(-)	
BMI, per kg/m^2	0.94 (0.89 – 1.0)	0.049	(-)	
Serum albumin, per g/dL	0.34 (0.21 – 0.56)	<0.001	0.36 (0.21 – 0.59)	<0.001
TLC, <1.5 ×10^9/L	1.8 (1.0 – 3.0)	0.040	1.6 (0.91 – 2.8)	0.10
Serum CRP, ≥1.0 mg/dL	2.5 (1.5 – 4.1)	<0.001	(-)	
Underlying pneumonia	1.9 (1.1 – 3.2)	0.02	(-)	

HR, hazard ratio; † Explanatory variables were selected using Akaike information criterion. AIC, Akaike information criterion; HR, hazard ratio; CI, confidence interval; L3-SMI, Skeletal muscle mass index at the third lumber level; BMI, body mass index; CRP, C-reactive protein; PNI, Onodera's prognostic nutritional index; WBC, white blood cell count; and TLC, total lymphocyte count.

(b) Cox proportional hazard analysis with variable selection method using P-value in univariate analysis of under 0.1 and 0.05

There was no difference in results between a P-value threshold of 0.05 and 0.1 because no variable had a P-value in the range of 0.05 to 0.1 in the univariate analysis.

	Univariate analysis		Multivariate analysis (AIC)	
Variables	HR (95% CI)	*P* value	Adjusted HR (95% CI)	*P* value
L3-SMI, low	2.8 (1.6 – 4.8)	<0.001	2.2 (1.2 – 4.3)	0.014
Sex, male	1.8 (1.0 – 3.3)	0.046	1.9 (0.97 – 3.7)	0.062
Age, per year	1.9 (1.2 – 3.2)	0.01	1.0 (0.99 – 1.0)	0.30
BMI, per kg/m^2	0.94 (0.89 – 1.0)	0.049	0.97 (0.90 – 1.0)	0.35
Serum albumin, per g/dL	0.34 (0.21 – 0.56)	<0.001	0.49 (0.27 – 0.89)	0.019
TLC, $<1.5 \times 10^9$/L	1.8 (1.0 – 3.0)	0.040	1.4 (0.81 – 2.5)	0.23
Serum CRP, ≥1.0 mg/dL	2.5 (1.5 – 4.1)	<0.001	1.6 (0.82 – 2.9)	0.18
Underlying pneumonia	1.9 (1.1 – 3.2)	0.02	1.0 (0.57 – 1.8)	0.96

* HR, hazard ratio; † Explanatory variables were selected using Akaike information criterion. AIC, Akaike information criterion; HR, hazard ratio; CI, confidence interval; L3-SMI, Skeletal muscle mass index at the third lumber level; BMI, body mass index; CRP, C-reactive protein; PNI, Onodera's prognostic nutritional index; WBC, white blood cell count; and TLC, total lymphocyte count.

(c) Cox proportional hazard analysis with variable selection method using P-value in univariate analysis of under 0.01

	Univariate analysis		Multivariate analysis (AIC) †	
Variables	HR (95% CI)	*P* value	Adjusted HR (95% CI)	*P* value
L3-SMI, low	2.8 (1.6 – 4.8)	<0.001	2.8 (1.6 – 5.0)	<0.001
Sex, male	1.8 (1.0 – 3.3)	0.046	(-)	
Age, per year	1.9 (1.2 – 3.2)	0.01	(-)	
BMI, per kg/m^2	0.94 (0.89 – 1.0)	0.049	(-)	
Serum albumin, per g/dL	0.34 (0.21 – 0.56)	<0.001	0.42 (0.24 – 0.73)	0.0020
TLC, <1.5 ×10^9/L	1.8 (1.0 – 3.0)	0.040	(-)	
Serum CRP, ≥1.0 mg/dL	2.5 (1.5 – 4.1)	<0.001	1.1 (0.98 – 1.2)	0.15
Underlying pneumonia	1.9 (1.1 – 3.2)	0.02	(-)	

HR, hazard ratio; † Explanatory variables were selected using Akaike information criterion. AIC, Akaike information criterion; HR, hazard ratio; CI, confidence interval; L3-SMI, Skeletal muscle mass index at the third lumber level; BMI, body mass index; CRP, C-reactive protein; PNI, Onodera's prognostic nutritional index; WBC, white blood cell count; and TLC, total lymphocyte count.

D. Sensitivity analysis for the covariate balancing propensity score-based analysis

We investigated the fluctuation of the main values of survival analysis when propensity score was used as a covariate instead of matching.

	Univariate analysis		Multivariate analysis	
Variables	HR (95% CI)	*P* value	HR adjusted for PS (95% CI)	*P* value
L3-SMI, low	2.6 (1.4 – 4.6)	0.001	2.2 (1.2 – 4.1)	0.007

* P-value was calculated by Cox proportional hazard model using two explanatory variables of L3-SMI and propensity score. The propensity score was calculated using the variables of ‘underlying pneumoniae (yes/no)’, ‘sex (male/female)’, ‘age (years)’, ‘serum albumin level (g/dL)’, ‘C-reactive protein level (mg/dL)’, ‘total lymphocyte count (/µL)’. HR, hazard ratio; CI, confidence interval; PS, propensity score; and L3-SMI, Skeletal muscle mass index at the third lumber level
